# Humidity Reduces Rapid and Distant Airborne Dispersal of Viable Viral Particles in Classroom Settings

**DOI:** 10.1021/acs.estlett.2c00243

**Published:** 2022-06-16

**Authors:** Antun Skanata, Fabrizio Spagnolo, Molly Metz, Davida S. Smyth, John J. Dennehy

**Affiliations:** † Biology Department, Queens College, The City University of New York, Flushing, New York 11367, United States; ‡ Department of Natural Sciences and Mathematics, Eugene Lang College of Liberal Arts at The New School, New York, New York 10011, United States; § Biology Doctoral Program, The Graduate Center, The City University of New York, New York, New York 10016, United States

**Keywords:** long-range transmission, airborne pathogens, aerosol generation, SARS-CoV-2, bacteriophage Phi6, ambient humidity

## Abstract

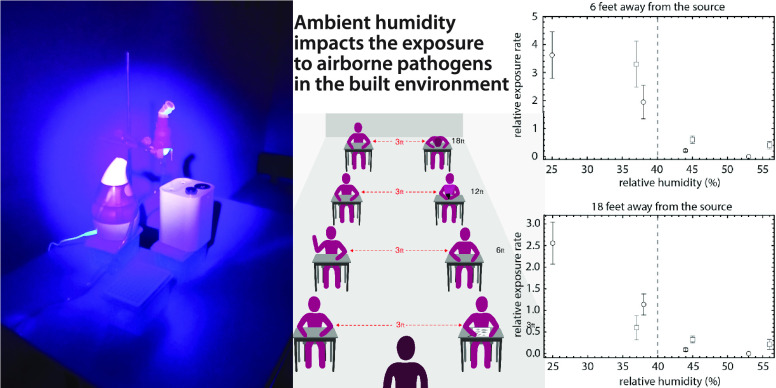

The transmission of airborne pathogens is considered to be the main route through which a number of known and emerging respiratory diseases infect their hosts. While physical distancing and mask wearing may help mitigate short-range transmission, the extent of long-range transmission in closed spaces where a pathogen remains suspended in the air remains unknown. We have developed a method to detect viable virus particles by using an aerosolized bacteriophage Phi6 in combination with its host *Pseudomonas phaseolicola*, which when seeded on agar plates acts as a virus detector that can be placed at a range of distances away from an aerosol-generating source. By applying this method, we consistently detected viable phage particles at distances of up to 18 feet away from the source within 15 min of exposure in a classroom equipped with a state of the art HVAC system and determined that increasing the relative humidity beyond 40% significantly reduces dispersal. Our method, which can be further modified for use with other virus/host combinations, quantifies airborne transmission in the built environment and can thus be used to set safety standards for room capacity and to ascertain the efficacy of interventions in closed spaces of specified sizes and intended uses.

## Introduction

Airborne transmission of human pathogens has been a driver of major outbreaks of known and novel respiratory diseases.^1^ It is now known that a number of contagious diseases can spread through a process of aerosolization, where an infected individual generates virus-carrying particles that can remain suspended in the air for long periods.^2,3^ While airborne transmission rarely occurs outdoors,^4^ indoor spaces may facilitate transmission via aerosols even while physically distancing.^5^ Experiments have shown that many viruses, including SARS-CoV-2, remain infectious for long periods when suspended in aerosols.^6^ As such, it is crucial to determine the distances and time scales over which pathogens can spread indoors and ask what types of shared space usage recommendations can be implemented to minimize future outbreaks.

**Figure 1:**
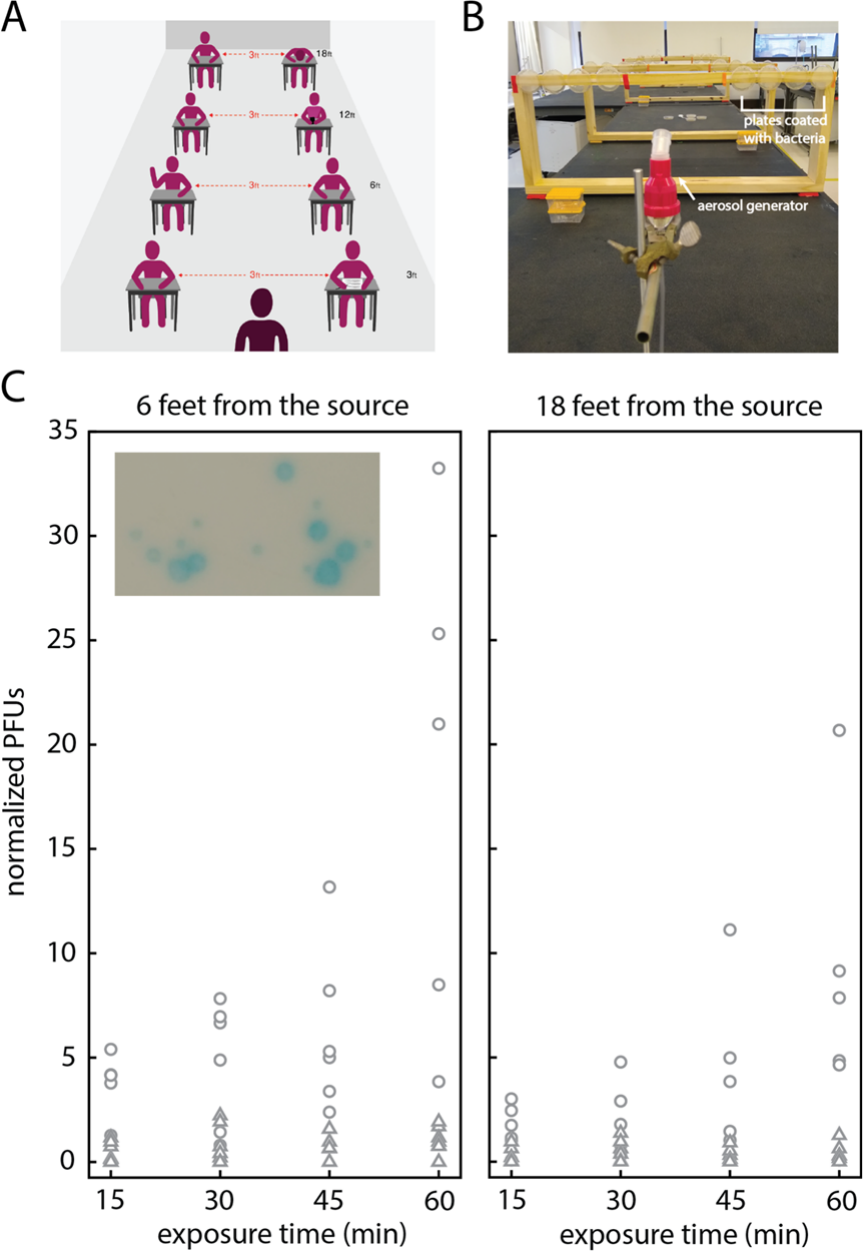
Experiments to determine the spreading of viable aerosolized pathogens in closed spaces. (A) Graphical representation of a typical classroom setting where physical distancing is enforced. (B) Layout of the experiment. Detectors are placed at specified distances away from a nebulizer (located in the front of the photo). At each location we placed four detectors, which we expose for increasing durations of time: 15, 30, 45, and 60 min total. (C) Plaque counts as a function of exposure time for detectors placed 6 feet away (left panel) and 18 feet away from the source (right panel). Data points correspond to data collected in two different rooms, in a total of seven independent experiments. Circles correspond to data collected at RH below 40%; triangles correspond to data points collected at RH above 40%. Counts are normalized to 4 × 10^7^ total phage released. Data at all distances are shown in Figure S1. Inset: Blue plaque morphology shows distinct size differences.

To parametrize viral dispersal in the built environment, we developed a method to detect viable virus particles in aerosols by using a bacterium *Pseudomonas syringae* pv *phaseolicola* genetically modified to produce LacZ-α, which serves as the host for a *LacZ-*β-marked bacteriophage, Phi6. Phi6 is a lipid-coated icosahedral bacteriophage commonly used as a proxy for human enveloped viruses, including SARS-CoV-2, due to its similar structure, size, and physiology.^7−9^ When plated on agar containing X-Gal and the LacZ-α expressing host, these phages produce easily identifiable blue plaques, which are lesions in the bacterial lawn where phages have lysed their hosts (Figure 1C, inset). X-Gal agar plates overlaid with soft agar containing the *P. phaseolicola* host and exposed for varying durations thus act as virus detectors, which can be placed at a range of distances from the aerosol-generating source of phage Phi6 (Figure 1B).

Here, we report two major results: (1) Aerosolized Phi6 particles can travel distances of 18 feet in under 15 min in a closed room equipped with an HVAC system operating above an industry recommended level.^10^ (2) Exposure to the aerosolized virus decreases with increases in relative humidity (RH), for the range of RH that naturally occurs in air-conditioned environments in our region. Specifically, we find that the exposure is significantly reduced when RH is above 40% at a temperature of (22.8 ± 0.2) °C. These results may be useful in determining the types of interventions capable of reducing or eliminating the exposure to airborne pathogens in shared closed spaces.

## Materials and Methods

### Preparation of Viral Surrogate and Host

A single colony of LacZ-α producing *P. phaseolicola* was added to 10 mL of lysogeny broth (LB) and incubated for 18 h with rotary shaking (220 rpm) at 25 °C.^11,12^ Then, 500 μL of the overnight culture was added to 50 mL of fresh LB supplemented with 200 μg/mL ampicillin and incubated for 18 h with rotary shaking (200 rpm) at 25 °C to provide overnight culture for soft agar overlay plates (200 μL overnight culture in 3 mL soft agar) that served as detectors for our experiments. To produce Phi6 lysate, 5 mL of stationary-phase culture was added to 200 mL fresh LB, along with 10 μL frozen phage stock when the culture reached exponential phase.^13^ Following 18 h incubation with shaking, phages were isolated by filtration through 0.22 μm filters (Durapore; Millipore, Bedford, MA). Phage particles per mL were quantified via serial dilution and plating according to standard methods (Supporting Information).^13^

### Generation of Aerosols

To generate aerosolized viral droplets, we introduced 10 mL of phage lysate diluted in LB into a medical grade nebulizer (Uni-HEART Lo-Flo Continuous Nebulizer, Westmed Inc.), which when connected to an air compressor (Westmed Model 0399) continually generates aerosols of 2–3 μm mass median aerodynamic diameter (MMAD), according to the manufacturer’s data and specifications. Compressor output is rated at 6 L/min, which is comparable to normal adult minute ventilation estimates.^14^ Aerosols of 2–3 μm are known to deposit in the nose, lungs, and bronchi in adults upon inhaling.^15^ Generation of aerosols in this size range has been associated with talking and coughing.^16^ The inherent variability of aerosol sizes generated by the nebulizer might effectively capture some of the variability in aerosols that are also produced by other common actions such as breathing, sneezing, exhaling, and talking softly.^16,17^

We diluted phage lysates to concentrations of approximately 10^8^ phage particles per mL and estimated titers used in each experiment by spot plating 10 μL aliquots at different dilutions on replicate plates. The titers we report in SI Tables S1, S2, and S3 are averages over three–five replicates. These titers led to consistently countable numbers of plaques across all distances, exposure times, and external conditions. Symptomatic individuals with coronavirus and influenza virus infections can shed from 10^2^ to 10^5^ viral particles over the course of 30 min.^18,19^ While different viruses might require a different inoculum size to successfully infect the human host, it has been shown that even a few virions of influenza A can generate infections.^20^

### Classroom Experiments

We arranged the placement of the source of aerosols and the detectors in such a way to consider the risks of spreading of airborne viruses in a classroom (Figure 1A), simulating a situation where mask wearing is not enforced. The mouth of the nebulizer and the centers of the detectors were at a height of 55 ± 1 in. from the floor, representing the height of a source and the breathing zone of hosts sitting around a long bench (Figure 1B). The detectors were located 3, 6, 12, and 18 feet away from the nebulizer to study the impact of airborne transmission in closed spaces. We placed two sets of four detectors that are laterally 3 feet apart while being approximately the same distance away from the source. Single detectors within each set were exposed to the aerosolized phage for progressively longer durations of time for up to 1 h (i.e., 15, 30, 45, and 60 min). Such placement of the detectors provides a replicate measurement within the same experiment. We repeated our experiments over multiple days in two different rooms at The New School, located on different floors, and whose climates are controlled by separate, independent HVAC systems. Our results are consistent among the two rooms despite the variation generated by changes in external conditions, differences in room size, placement of air returns, and other sources (Supporting Information and Figure S2).

## Results and Discussion

### Aerosolized Phage Can Spread over Large Distances

We performed our experiments in two classrooms at The New School in New York City, equipped with a state of the art HVAC system with filtration corresponding to two rows of filters (prefilter M8 and final filter M14), which continuously operates at about 15 room air changes per hour (private correspondence). Yet, we consistently observed plaque-forming units (PFUs) on plates at distances of up to 18 feet away from the nebulizer and exposed for a duration of 15 min from the start of aerosol generation (Figure 1C). We found that the exposure to aerosolized phage particles at 18 feet away from the source shows on average a modest 1.6 times reduction when compared to the exposure observed at 6 feet over the same durations (Figure 1C).

### Humidity Impacts the Spread of Aerosolized Phage

We further found experimental evidence that the humidity impacts the spread and therefore the exposure to aerosolized phage. Figure 1C shows the data we collected across two rooms and a range of external conditions. The ambient temperature was consistently maintained by the HVAC system in the range 22.5–23 °C across all experiments, with temperatures fluctuating on average below 1% (Figures S3 and S4). However, we noticed an apparent difference in the data when we aligned the PFU counts with humidity recordings. Circles in Figure 1C that are dispersed across a range of PFUs show normalized counts collected at RH below 40%, while triangles, which are located in a narrow band at very low PFU counts, correspond to data collected at RH higher than 40%.

**Figure 2:**
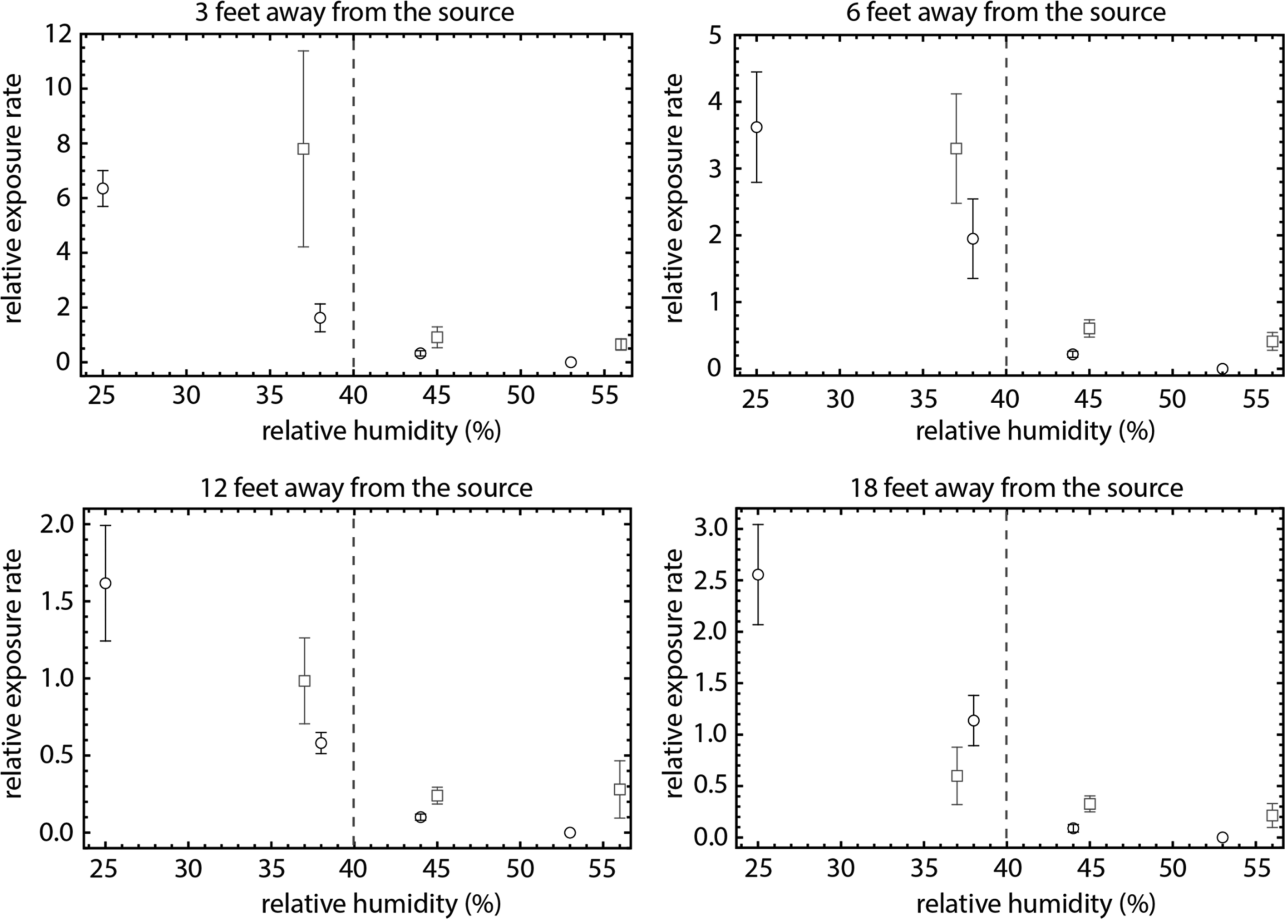
Exposure to aerosolized pathogens decreases with increasing relative humidity. Panels correspond to exposure at predetermined distances away from the source. Points are averages of PFUs normalized to 4 × 10^7^ total phage released and expressed as a rate per 15 min exposure for a particular experiment, as a function of humidity, in two classrooms at The New School (rectangles, Room 300; circles, Room 400). Error bars are ± SE. RH has a margin of error ±2% according to manufacturer’s specifications. Dashed vertical line at 40% relative humidity visually separates regions of high exposure (RH below 40%) and low exposure (RH above 40%).

Figure 2 shows the rate at which PFUs are generated per 15 min exposure time as a function of RH and distance from the nebulizer. For plates that were exposed for longer than 15 min, we consider the average rate of PFU formation in a 15 min time window. At all distances, we find that increasing RH above 40% results in a substantial decrease in exposure rates. These results are consistent among the two classrooms in which we conducted our experiments, represented with circles and squares. Experimental evidence suggests similar dependence on humidity for transmission of influenza.^21−23^

### Discussion

Our experiments found that aerosolized phage particles can travel large distances in short amounts of time in closed spaces that are equipped with HVAC systems. We determined that the exposure to the surrogate virus significantly decreases with increases in RH. A question remains whether this decrease is a result of loss of viability at a higher humidity^21,24,25^ or a result of processes that limit the dispersal of virus-carrying particles, which may include reduced droplet evaporation and increased surface deposition at higher RH.^2^ To address this question, we performed additional experiments with the detectors placed horizontally on the bench surface at distances of 3, 6, 12, and 18 feet away from the nebulizer. Such placement of the detectors measures horizontal surface deposition rates at those locations. Figure S5 shows that the rate of deposition on horizontal surfaces decreases with increasing RH at distances of 3 feet and beyond, suggesting that if the surface deposition rates are increased, those increases are limited to a radius within 3 feet away from the source. We also performed control experiments in a room where the HVAC system was turned off (Figure S5) and found no significant differences in exposure rates.

These results led us to speculate that, in closed spaces where levels of humidity higher than 40% cannot be achieved or controlled, a relatively inexpensive personal humidifier might provide an individualized layer of protection. We placed portable battery-operated personal humidifiers at one of the two replicate locations at different distances away from the source, and compared the exposure in the same room, under the same environmental conditions, of a set of detectors with and without a personal humidifier located at a distance of 6 in (15 cm) away from and in front of the detector plates (Figure S6). The detectors located next to personal humidifiers showed a decrease in exposure to airborne pathogens.

Observed plaques in our experiments show a range of sizes (Figure 1C, inset). Insertion of a genetic marker to phage Phi6 is known to induce mutations that may subsequently resolve in smaller plaque sizes.^13^ Another possibility is that since there exist variations in pathogen load when sampling small volumes for aerosolization, larger plaques may have been initially formed from particles that carry multiple phage. Since we do not distinguish between a plaque generated by a single phage or multiple phage, we count each plaque as a single infection event. The true integrated exposure to aerosolized phage might be higher than our plates suggest.

Respiratory droplets span a range of sizes (from 1 μm to 1 mm), which depend on the respiratory mode (i.e., breathing, speaking, coughing, sneezing, etc.). Any individual will likely generate particles of all sizes within that range over the course of an hour. Larger particles will be most relevant for short-range transmission, which may be mitigated through physical distancing, and which we enforced by placing the detectors 3 feet or more away from the source. Therefore, the remaining problem is of the long-range transmission, for which particles of size below 5 μm are particularly relevant. We have chosen to work with particles of about 2–3 μm MMAD to address the long-range transmission, which is thought to be the driver of the COVID-19 pandemic, but is also relevant for a host of airborne pathogens, including tuberculosis,^26^ measles,^27^ influenza,^28,29^ and others.^30,31^

We applied our method to experimentally determine humidity-transmission curves in the built environment, which depend on environmental factors and the biological properties of the virus. Studies of survival and infectivity of a number of human pathogens show a strong dependence on ambient humidity.^32^ Many enveloped viruses, including Influenza A^31,33^ and SARS-CoV-2,^24^ can survive better at low RH, followed by a decrease in viability at intermediate RH, and subsequent increase at high RH.^32^ The infectivity of phage Phi6 suspended in aerosols and droplets shows a similar pattern with the increase in RH at room temperatures.^25^ Our results are consistent with this body of work and suggest that maintaining RH in this intermediate range of 40%–60% can significantly reduce transmission for a number of pathogens. Viral loads of aerosols and their potential to spur infection will depend on the pathogen; therefore, our results by using a phage do not reflect exposure risk to a particular pathogen. We also note that breathing is an active process that involves sampling the air from a surrounding volume, whereas our plates detect the rates of aerosol deposition on vertical surfaces. Establishing a mapping between the counts of PFUs on plates and dosage through inhalation can be achieved by using an air sampler in parallel to the static plates and is left for future work.

Long-range transmission risks in closed ventilated spaces can strongly depend on the environment. In this work, we limited the real-life environment to the furthest extent possible, i.e., by controlling the temperature, minimizing airflow disturbances, and excluding heat sources that would simulate the disturbances due to stationary hosts. All of these factors could contribute to the transmission curves in nontrivial ways; our method can be applied to such and other more complex settings. The placement of the source and the detectors can simulate various situations in which transmission can occur and are not constrained to align vertically or horizontally. For example, in a hospital setting, the nebulizer can be placed at a height of the patient, while the detectors can be set at various locations and heights within the room to model transmission risks to the staff and visitors.

In this work, we found that exposure to airborne pathogens may occur at any conceivable distance, regardless of the length of time a person is present in the room. Current room capacity designations consider room surface area, placement of physical barriers, and air ventilation and filtration capabilities.^34^ However, exposure risks to airborne pathogens in closed spaces might not be dominated by space considerations alone. Our results show that relative humidity plays a major role in long-range transmission risks. It is widely recognized that air flows in a closed room are dependent on the specifics of the space and the activity of its occupants. Efforts, which include designing ventilation systems that adjust to the number of occupants and their activity^35^ and assessing the exposure on the basis of activity, distancing, mask wearing, and filtration efficiency,^36^ are currently underway.

The disparity between the conditions within which people of different socio-economic status live and work was a major driver of the COVID-19 pandemic.^37,38^ Many office buildings and private institutions may have the funds required to implement a range of exposure-reducing interventions, while public institutions or shared-housing complexes may not. Here, we developed a method to measure the transmission of airborne pathogens in closed spaces, which is portable (can be produced in a wet lab and ported to site), relatively inexpensive, and can be deployed to a variety of spaces with different configurations and intended uses. This method, supplemented with epidemiological modeling, could form a basis for inferring data-driven recommendations on room capacity and risk-reducing interventions that can be specific to the space and its intended occupants.

## Supporting Information Available

Supporting methods, Figures S1−S6, and data tables (PDF)

## References

[ref1] Leung N. H. L. Transmissibility and Transmission of Respiratory Viruses. Nat. Rev. Microbiol. 2021, 19 (8), 528–545. 10.1038/s41579-021-00535-6. 33753932 PMC7982882

[ref2] Wells W. F. On Air-Borne Infection*: Study II. Droplets And Droplet Nuclei. Am. J. Epidemiol. 1934, 20 (3), 611–618. 10.1093/oxfordjournals.aje.a118097.

[ref3] van Doremalen N.; Bushmaker T.; Morris D. H.; Holbrook M. G.; Gamble A.; Williamson B. N.; Tamin A.; Harcourt J. L.; Thornburg N. J.; Gerber S. I.; Lloyd-Smith J. O.; de Wit E.; Munster V. J. Aerosol and Surface Stability of SARS-CoV-2 as Compared with SARS-CoV-1. N. Engl. J. Med. 2020, 382 (16), 1564–1567. 10.1056/NEJMc2004973. 32182409 PMC7121658

[ref4] Bulfone T. C.; Malekinejad M.; Rutherford G. W.; Razani N. Outdoor Transmission of SARS-CoV-2 and Other Respiratory Viruses: A Systematic Review. J. Infect. Dis. 2021, 223 (4), 550–561. 10.1093/infdis/jiaa742. 33249484 PMC7798940

[ref5] Mendell M. J.; Fisk W. J.; Kreiss K.; Levin H.; Alexander D.; Cain W. S.; Girman J. R.; Hines C. J.; Jensen P. A.; Milton D. K.; Rexroat L. P.; Wallingford K. M. Improving the Health of Workers in Indoor Environments: Priority Research Needs for a National Occupational Research Agenda. Am. J. Public Health 2002, 92 (9), 1430–1440. 10.2105/AJPH.92.9.1430. 12197969 PMC1447254

[ref6] Fears A. C.; Klimstra W. B.; Duprex P.; Hartman A.; Weaver S. C.; Plante K. C.; Mirchandani D.; Plante J. A.; Aguilar P. V.; Fernández D.; Nalca A.; Totura A.; Dyer D.; Kearney B.; Lackemeyer M.; Bohannon J. K.; Johnson R.; Garry R. F.; Reed D. S.; Roy C. J. Comparative Dynamic Aerosol Efficiencies of Three Emergent Coronaviruses and the Unusual Persistence of SARS-CoV-2 in Aerosol Suspensions. medRxiv Preprint, 2020. 10.1101/2020.04.13.20063784.

[ref7] Adcock N. J.; Rice E. W.; Sivaganesan M.; Brown J. D.; Stallknecht D. E.; Swayne D. E. The Use of Bacteriophages of the Family Cystoviridae as Surrogates for H5N1 Highly Pathogenic Avian Influenza Viruses in Persistence and Inactivation Studies. J. Environ. Sci. Health Part A Tox. Hazard. Subst. Environ. Eng. 2009, 44 (13), 1362–1366. 10.1080/10934520903217054. 20183493

[ref8] Aquino de Carvalho N.; Stachler E. N.; Cimabue N.; Bibby K. Evaluation of Phi6 Persistence and Suitability as an Enveloped Virus Surrogate. Environ. Sci. Technol. 2017, 51 (15), 8692–8700. 10.1021/acs.est.7b01296. 28657725

[ref9] Fedorenko A.; Grinberg M.; Orevi T.; Kashtan N. Survival of the Enveloped Bacteriophage Phi6 (a Surrogate for SARS-CoV-2) in Evaporated Saliva Microdroplets Deposited on Glass Surfaces. Sci. Rep. 2020, 10 (1), 22419 10.1038/s41598-020-79625-z. 33376251 PMC7772334

[ref10] Stewart E. J.; Schoen L. J.; Mead K.; Olmsted R. N.; Sekhar C.; Vernon W.; Pantelic J.; Li Y.; Sultan Z. M.; Conlan W. ASHRAE Position Document on Infectious Aerosols; ASHRAE, 2020.

[ref11] Onodera S.; Qiao X.; Gottlieb P.; Strassman J.; Frilander M.; Mindich L. RNA Structure and Heterologous Recombination in the Double-Stranded RNA Bacteriophage Phi 6. J. Virol. 1993, 67 (8), 4914–4922. 10.1128/jvi.67.8.4914-4922.1993. 8331732 PMC237879

[ref12] Froissart R.; Wilke C. O.; Montville R.; Remold S. K.; Chao L.; Turner P. E. Co-Infection Weakens Selection Against Epistatic Mutations in RNA Viruses. Genetics 2004, 168 (1), 9–19. 10.1534/genetics.104.030205. 15454523 PMC1448111

[ref13] Ford B. E.; Sun B.; Carpino J.; Chapler E. S.; Ching J.; Choi Y.; Jhun K.; Kim J. D.; Lallos G. G.; Morgenstern R.; Singh S.; Theja S.; Dennehy J. J. Frequency and Fitness Consequences of Bacteriophage Φ6 Host Range Mutations. PLoS One 2014, 9 (11), e113078 10.1371/journal.pone.0113078. 25409341 PMC4237377

[ref14] Beals J. A.; Funk L. M.; Fountain R.; Sedman R. Quantifying the Distribution of Inhalation Exposure in Human Populations: Distribution of Minute Volumes in Adults and Children. Environ. Health Perspect. 1996, 104 (9), 974–979. 10.1289/ehp.96104974. 8899377 PMC1469465

[ref15] Bates D. V.; Fish B. R.; Hatch T. F.; Mercer T. T.; Morrow P. E. Deposition and Retention Models for Internal Dosimetry of the Human Respiratory Tract. Task Group on Lung Dynamics. Health Phys. 1966, 12 (2), 173–207. 5916786

[ref16] Morawska L. Droplet Fate in Indoor Environments, or Can We Prevent the Spread of Infection?. Indoor Air 2006, 16 (5), 335–347. 10.1111/j.1600-0668.2006.00432.x. 16948710

[ref17] Pöhlker M. L.; Krüger O. O.; Förster J.-D.; Berkemeier T.; Elbert W.; Fröhlich-Nowoisky J.; Pöschl U.; Pöhlker C.; Bagheri G.; Bodenschatz E.; Huffman J. A.; Scheithauer S.; Mikhailov E. Respiratory Aerosols and Droplets in the Transmission of Infectious Diseases. arXiv Preprint, arXiv:2103.01188, 2021. 10.48550/arXiv.2103.01188.

[ref18] Yan J.; Grantham M.; Pantelic J.; Bueno de Mesquita P. J.; Albert B.; Liu F.; Ehrman S.; Milton D. K.; Adamson W.; Beato-Arribas B.; Bischoff W.; Booth W.; Cauchemez S.; Ehrman S.; Enstone J.; Ferguson N.; Forni J.; Gilbert A.; Grantham M.; Grohskopf L.; Hayward A.; Hewitt M.; Kang A.; Killingley B.; Lambkin-Williams R.; Mann A.; Milton D.; Nguyen-Van-Tam J.; Noakes C.; Oxford J.; Palmarini M.; Pantelic J.; Wang J.; Bennett A.; Cowling B.; Monto A.; Tellier R. Infectious Virus in Exhaled Breath of Symptomatic Seasonal Influenza Cases from a College Community. Proc. Natl. Acad. Sci. U. S. A. 2018, 115 (5), 1081–1086. 10.1073/pnas.1716561115. 29348203 PMC5798362

[ref19] Leung N. H. L.; Chu D. K. W.; Shiu E. Y. C.; Chan K.-H.; McDevitt J. J.; Hau B. J. P.; Yen H.-L.; Li Y.; Ip D. K. M.; Peiris J. S. M.; Seto W.-H.; Leung G. M.; Milton D. K.; Cowling B. J. Respiratory Virus Shedding in Exhaled Breath and Efficacy of Face Masks. Nat. Med. 2020, 26 (5), 676–680. 10.1038/s41591-020-0843-2. 32371934 PMC8238571

[ref20] Alford R. H.; Kasel J. A.; Gerone P. J.; Knight V. Human Influenza Resulting from Aerosol Inhalation. Proc. Soc. Exp. Biol. Med. 1966, 122 (3), 800–804. 10.3181/00379727-122-31255. 5918954

[ref21] Harper G. J. Airborne Micro-Organisms: Survival Tests with Four Viruses. Epidemiol. Infect. 1961, 59 (4), 479–486. 10.1017/S0022172400039176. PMC2134455 13904777

[ref22] Noti J. D.; Blachere F. M.; McMillen C. M.; Lindsley W. G.; Kashon M. L.; Slaughter D. R.; Beezhold D. H. High Humidity Leads to Loss of Infectious Influenza Virus from Simulated Coughs. PLoS One 2013, 8 (2), e57485 10.1371/journal.pone.0057485. 23460865 PMC3583861

[ref23] Marr L. C.; Tang J. W.; Van Mullekom J.; Lakdawala S. S. Mechanistic Insights into the Effect of Humidity on Airborne Influenza Virus Survival, Transmission and Incidence. J. R. Soc. Interface 2019, 16 (150), 20180298 10.1098/rsif.2018.0298. 30958176 PMC6364647

[ref24] Morris D. H.; Yinda K. C.; Gamble A.; Rossine F. W.; Huang Q.; Bushmaker T.; Fischer R. J.; Matson M. J.; Van Doremalen N.; Vikesland P. J.; Marr L. C.; Munster V. J.; Lloyd-Smith J. O. Mechanistic Theory Predicts the Effects of Temperature and Humidity on Inactivation of SARS-CoV-2 and Other Enveloped Viruses. eLife 2021, 10, e65902 10.7554/eLife.65902. 33904403 PMC8277363

[ref25] Prussin A. J.; Schwake D. O.; Lin K.; Gallagher D. L.; Buttling L.; Marr L. C. Survival of the Enveloped Virus Phi6 in Droplets as a Function of Relative Humidity, Absolute Humidity, and Temperature. Appl. Environ. Microbiol. 2018, 84 (12), e00551 10.1128/AEM.00551-18. 29625986 PMC5981065

[ref26] Kenyon T. A.; Valway S. E.; Ihle W. W.; Onorato I. M.; Castro K. G. Transmission of Multidrug-Resistant Mycobacterium Tuberculosis during a Long Airplane Flight. N. Engl. J. Med. 1996, 334 (15), 933–938. 10.1056/NEJM199604113341501. 8596593

[ref27] Remington P. L.; Hall W. N.; Davis I. H.; Herald A.; Gunn R. A. Airborne Transmission of Measles in a Physician’s Office. JAMA 1985, 253 (11), 1574–1577. 10.1001/jama.1985.03350350068022. 3974036

[ref28] Schulman J. L.; Kilbourne E. D. Airborne Transmission of Influenza Virus Infection in Mice. Nature 1962, 195 (4846), 1129–1130. 10.1038/1951129a0. 13909471

[ref29] Shaman J.; Kohn M. Absolute Humidity Modulates Influenza Survival, Transmission, and Seasonality. Proc. Natl. Acad. Sci. U. S. A. 2009, 106 (9), 3243–3248. 10.1073/pnas.0806852106. 19204283 PMC2651255

[ref30] Tamerius J. D.; Shaman J.; Alonso W. J.; Bloom-Feshbach K.; Uejio C. K.; Comrie A.; Viboud C. Environmental Predictors of Seasonal Influenza Epidemics across Temperate and Tropical Climates. PLOS Pathog. 2013, 9 (3), e1003194 10.1371/journal.ppat.1003194. 23505366 PMC3591336

[ref31] Lowen A. C.; Mubareka S.; Steel J.; Palese P. Influenza Virus Transmission Is Dependent on Relative Humidity and Temperature. PLOS Pathog. 2007, 3 (10), e151 10.1371/journal.ppat.0030151. PMC2034399 17953482

[ref32] Yang W.; Marr L. C. Mechanisms by Which Ambient Humidity May Affect Viruses in Aerosols | Applied and Environmental Microbiology. Appl. Environ. Microbiol. 2012, 78 (19), 6781–6788. 10.1128/AEM.01658-12. 22820337 PMC3457514

[ref33] Niazi S.; Short K. R.; Groth R.; Cravigan L.; Spann K.; Ristovski Z.; Johnson G. R. Humidity-Dependent Survival of an Airborne Influenza A Virus: Practical Implications for Controlling Airborne Viruses. Environ. Sci. Technol. Lett. 2021, 8 (5), 412–418. 10.1021/acs.estlett.1c00253.

[ref34] Community, Work, and School: Ventilation in Buildings. Centers for Disease Control and Prevention. https://www.cdc.gov/coronavirus/2019-ncov/community/ventilation.html (accessed 2021–06–18).

[ref35] Morawska L.; Allen J.; Bahnfleth W.; Bluyssen P. M.; Boerstra A.; Buonanno G.; Cao J.; Dancer S. J.; Floto A.; Franchimon F.; Greenhalgh T.; Haworth C.; Hogeling J.; Isaxon C.; Jimenez J. L.; Kurnitski J.; Li Y.; Loomans M.; Marks G.; Marr L. C.; Mazzarella L.; Melikov A. K.; Miller S.; Milton D. K.; Nazaroff W.; Nielsen P. V.; Noakes C.; Peccia J.; Prather K.; Querol X.; Sekhar C.; Seppänen O.; Tanabe S.; Tang J. W.; Tellier R.; Tham K. W.; Wargocki P.; Wierzbicka A.; Yao M. A Paradigm Shift to Combat Indoor Respiratory Infection. Science 2021, 372 (6543), 689–691. 10.1126/science.abg2025. 33986171

[ref36] COV-IRT Member Signature Science LLC Releases Update to Its SARS-CoV-2 Exposure Assessment Tool, CEAT. COV-IRT. https://www.cov-irt.org/exposure-assessment-tool/ (acessed June 2022).

[ref37] Smyth D. COVID-19, Ebola, Measles: Achieving Sustainability in the Era of Emerging and Reemerging Infectious Diseases. Environ. Sci. Policy Sustain. Dev. 2020, 62 (6), 31–40. 10.1080/00139157.2020.1820295.

[ref38] Artiga S.; Corallo B.; Pham O. Racial Disparities in COVID-19: Key Findings from Available Data and Analysis; KFF, August 17, 2020.

