# Supplementary material for: Humidity Reduces Rapid and Distant Airborne Dispersal of Viable Viral Particles in Classroom Settings
Source: Environ Sci Technol Lett. 2022 Jun 16;9(7):632–7. doi: 10.1021/acs.estlett.2c00243 (PMC9344459; doi:10.1021/acs.estlett.2c00243)
Supplement: Supplementary file 1 — ez2c00243_si_001.pdf [file ez2c00243_si_001.pdf]

Supplementary Information

Humidity Reduces Rapid and Distant Airborne Dispersal of Viable Viral Particles in Classroom Settings

Antun Skanata*[a^], Fabrizio Spagnolo*[a@], Molly Metz [b], Davida S. Smyth[#b$], and John J. Dennehy[#a,c]

Affiliations
[a] Biology Department, Queens College, The City University of New York, New York; [b] Department of Natural Sciences and Mathematics, Eugene Lang College of Liberal Arts at The New School, New York; [c] Biology Doctoral Program, The Graduate Center, The City University of New York, New York

^ Current affiliation: Department of Physics, Syracuse University, Syracuse, NY
@ Current affiliation: Department of Biological & Environmental Sciences, Long Island University-Post, Brookville, NY
$ Current affiliation: Department of Life Sciences, Texas A&M University-San Antonio, Texas

# Address correspondence to John J. Dennehy, john.dennehy@qc.cuny.edu, Biology Department, Queens College, 65-30 Kissena Boulevard, Flushing, NY 11367, USA, and Davida S. Smyth, davida.smyth@tamusa.edu, Department of Life Sciences, Texas A&M University-San Antonio, One University Way, San Antonio, TX, 78224

Methods

Agar Preparation

LB medium (Miller Formula -Tryptone 10g/L, Yeast extract 5g/L, Sodium Chloride 10g/L) (BD Difco) was prepared according to the manufacturer's instructions. LB agar plates were supplemented with 200 µg/mL 5-bromo-4-chloro-3-indolyl-β-D-galactopyranoside (X-Gal) and 200 µg/mL ampicillin (Fisher Scientific). LB was also supplemented with 200 µg/mL ampicillin (Fisher Scientific). Plates were poured the night before and kept in the dark to cool. Soft agar was prepared using LB supplemented with agar (7g per liter). 3 mL of molten soft agar was aliquoted into 13 mm glass test tubes and maintained at 48℃ in a heating block. 200 µL of an overnight culture of *Pseudomonas syringae pv phaseolicola* was added, the contents vortexed gently and poured on top of an LB agar plate. For each batch of *P. phaseolicola* seeded plates, 2 plates were placed in the incubator as controls to assay for contamination.

Determination of Titers

Phage lysate was produced as previously described (Ref. 13 in the main text) and stored in glass bottles at 4℃ until use. Before each experiment, the titer was determined using a spot method, which provides a measure of sensitivity of the detector to phage particles being deposited on its surface. Lysate was serially diluted from 10^{-1} to 10^{-8} in LB. 10 µl of each dilution was spotted onto a seeded plate. The spotted plates were incubated for two days at 25℃. The titer was determined using the formula

Number of plaques * (1,000 µL/mL) / 10 µL / dilution factor = PFU/mL

Choice and Calibration of Nebulizers

Several brands of commercially available nebulizers were examined for their capability to resemble what is known about simulating breathing and coughing and we chose Uni-HEART™ Lo-Flo Continuous Nebulizers from Westmed Inc. owing to their ability to generate aerosols in the range of 2 to 3 µm. We noticed variation in the rate of aerosolization between individual nebulizer units. We labeled all of our nebulizer units and paired them with dedicated compressors. To calibrate the rate of nebulization, we ran a number of nebulizers in parallel for one hour and periodically monitored their operation. For these runs we used 10 mL of phosphate buffered saline (PBS; Fisher Scientific) and measured the remaining volume after one hour. For our experiments we chose the nebulizers which contained similar volumes of liquid after calibration. We note the rate of nebulization depends on the media used. The nebulizers we used for the experiments expelled 5.6 ± 0.8 mL of lysate in one hour.

Plate placement

The plates and nebulizer were placed on benches that are 37 inches from the floor. Plates were mounted to custom-made wooden stands with double-sided mounting tape. The center of the plates was at a height of 17.5 inches (room 300) and 19 inches (room 400) above the bench owing to differences in the height of the stands used in each room. The nebulizer was held aloft using a retort stand and clamp such that the mouth opening of the nebulizer was in line with the center of the agar plates. Air ducts are located to the sides of the bench as shown in Fig. S2. Duct openings are located at a height of 6.75 ft above the surface of the bench and 29 in beneath the ceiling. The ceiling height in both rooms is 12 ft; dimensions of the respective rooms and their layouts are shown in Fig. S2. Air returns are located on the wall adjacent to the doors, approximately in line with the orientation of the benches on which experiments were done. In room 300 the nebulizer was at the opposite end of the air return and in room 400 it was below and next to the air return (see Fig. S2), which allowed us to test two orientations of the experiment with respect to the overall direction of the airflow.

Temperature and humidity control

Each room has a dedicated Aircuity system which adjusts the flow rate according to CO_2 levels. From room dimensions (Fig. S2) and HVAC specifications that we obtained from facilities management at The New School we estimated the airflow rate in the room per air duct to be in the range of 300-350 CFM (cubic feet per minute). The location of the air ducts in each room is shown in Fig S2. Since there were no occupants in the rooms in which we performed the experiments (including a length of time prior to start of the experiments, and excluding a length of time required for setup and periodic retrieval of the plates), we expect that on average CO_2 levels remained constant and therefore airflows did not significantly change during data collection. Figs S3 and S4, which show small fluctuations in temperature and humidity for the duration of our experiments, support this conclusion. Somewhat larger fluctuations in humidity can be attributed to lower accuracy of readings, since temperature readings were accurate up to 0.2℃ and relative humidity up to 2%. We also tested for changes in humidity recordings by placing two devices at 9 feet away from the source and at 15 feet away from the source and did not observe significant differences. There is a possibility that due to the process of nebulization, a locally higher humidity is achieved within 3 feet away from the source, which was a cutoff distance in our experiments.

Experimental setup

Each plate was labelled with the date, room number, distance, exposure time and location of the plate with respect to the nebulizer. While the plates were being set up, each nebulizer was started without liquid to allow residual alcohol from cleaning (see below) to evaporate. The humidity and temperature was monitored using Govee Model:H5074 devices (accuracy ± 0.2℃ and ± 2% RH) placed on the bench surface at a distance 9 feet away from the nebulizer beginning 30 min before the experiments began to establish a baseline. Figures S3 and S4 show temperature and humidity charts for each individual experiment.

To start an experiment, we added 10 mL of phage lysate to the nebulizer, removed the lids from the plates and started the compressor. At the 15, 30, 45 and 60 minute marks, an experimenter entered the room, covered and removed a set of plates from the room. After all the plates were removed, the compressor was turned off.

The plates were incubated for 2-4 days at 25℃. Plaques were counted after two days of incubation after which they were moved to the bench. We did not observe additional plaques forming by the 4th day. After the experiment, the nebulizer was detached from the compressor and the remaining liquid removed using a thin tube attached to a syringe. The liquid was removed to a 15 mL conical tube and the volume was recorded. The extracted liquid was kept at room temperature for several days to monitor for contamination while the plates from the experiment were incubating. After each use, the nebulizers were washed in deionized water, the water removed, and the unit sprayed with 70% ethanol. The remaining alcohol was removed with a syringe and allowed to dry out before the next experiment. The nebulizers were replaced after 5-10 uses, after they started to leak or if they showed signs of contamination.

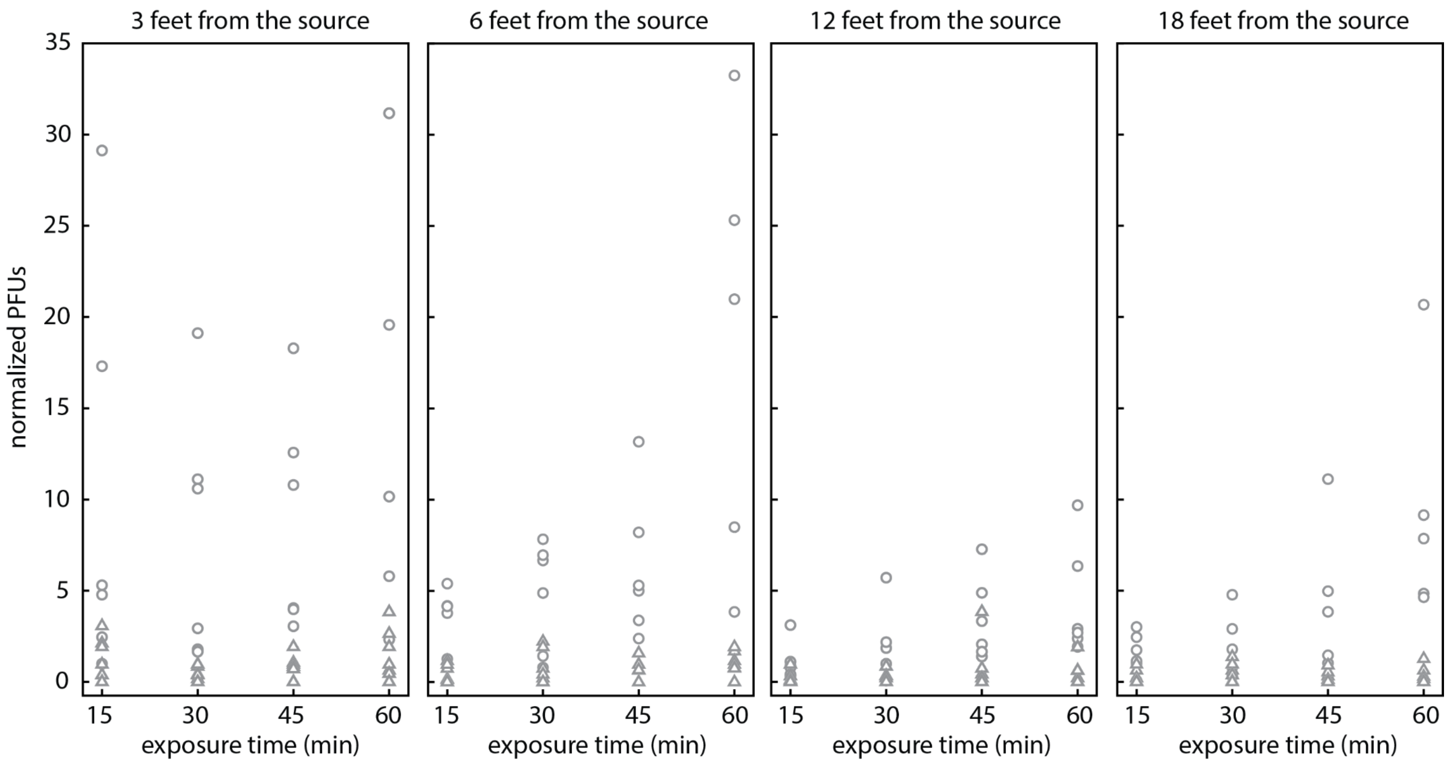

Figure S1: Plaque counts as a function of exposure time for detectors placed at all distances away from the source. Circles - data collected at relative humidities below 40%, triangles - data collected at relative humidities above 40%. Counts are normalized to 4×10^7 total phage released.

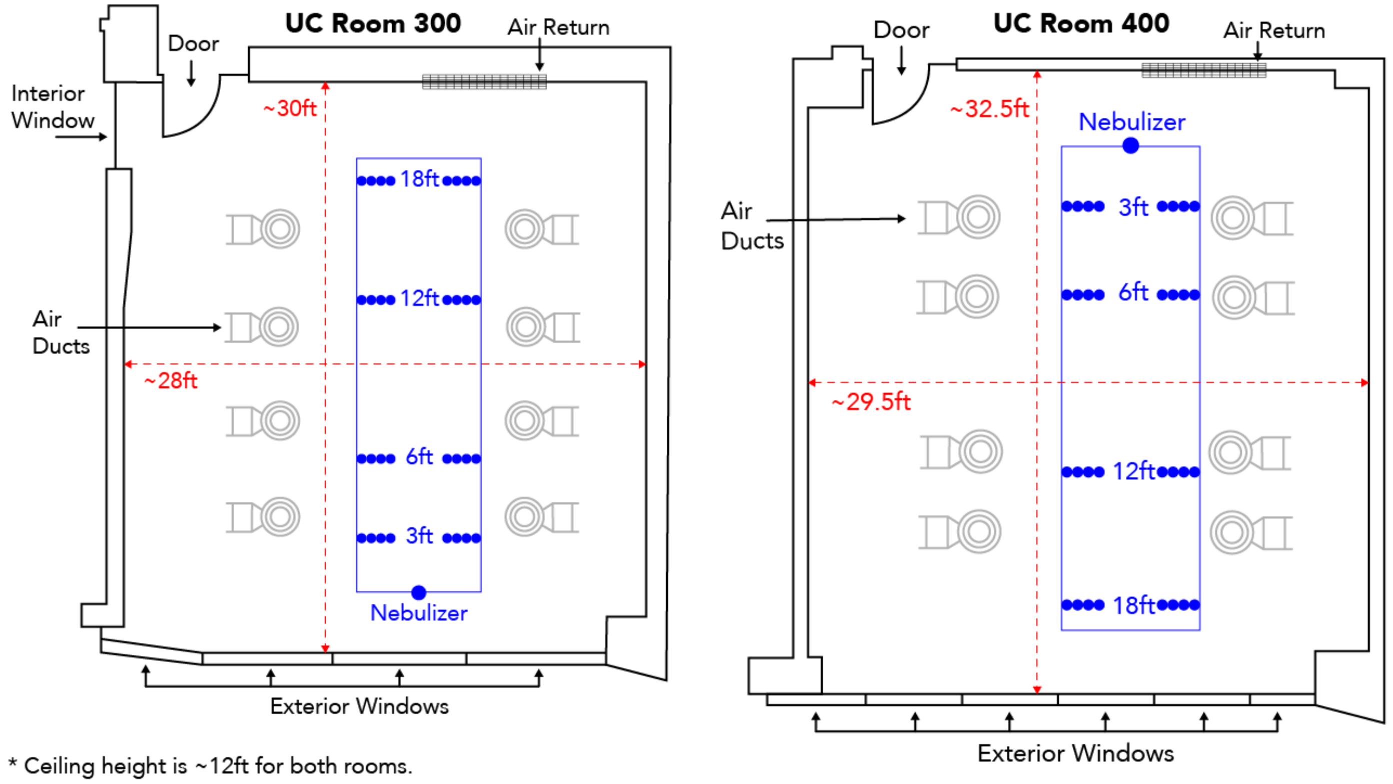

Figure S2: Floor plans of rooms 300 & 400 with approximate dimensions, placement of air ducts, air return, and their relation to the experimental setup.

Room **300** temperature and humidity recordings

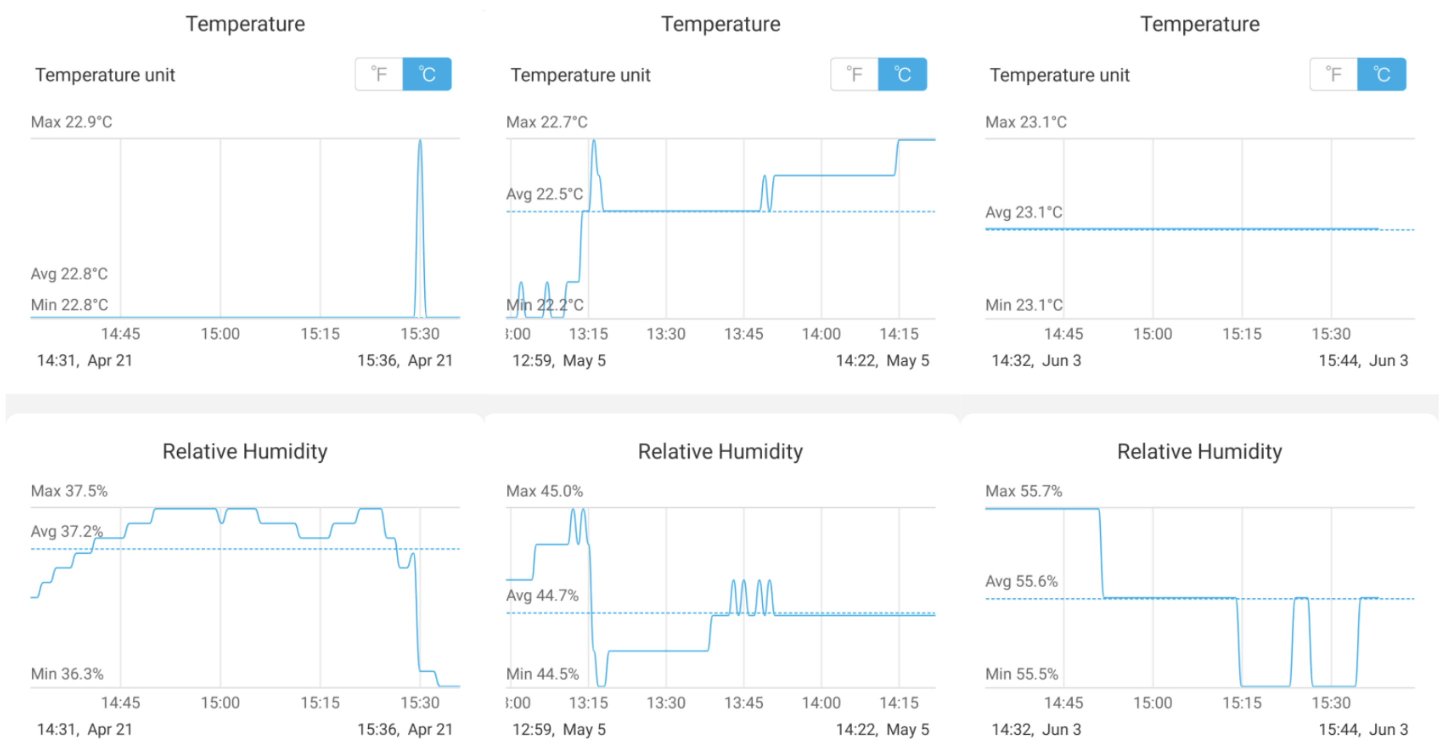

Figure S3: Temperature and humidity data for Room 300

Room **400** temperature and humidity recordings

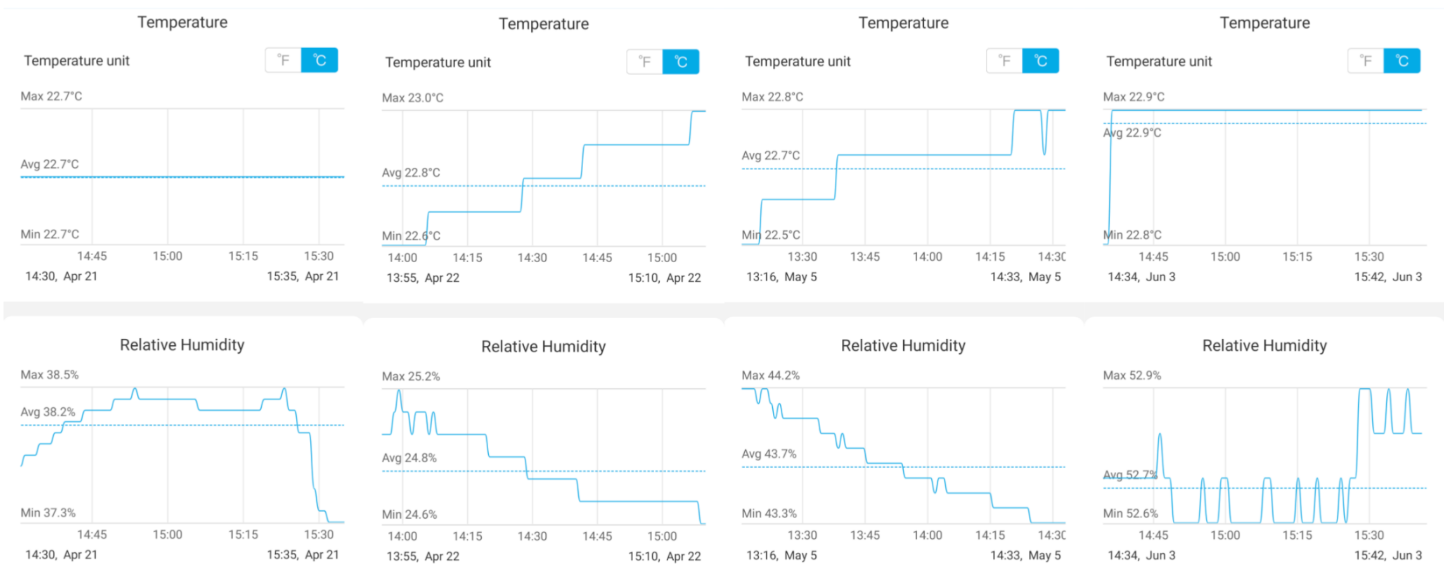

Figure S4: Temperature and humidity data for Room 400

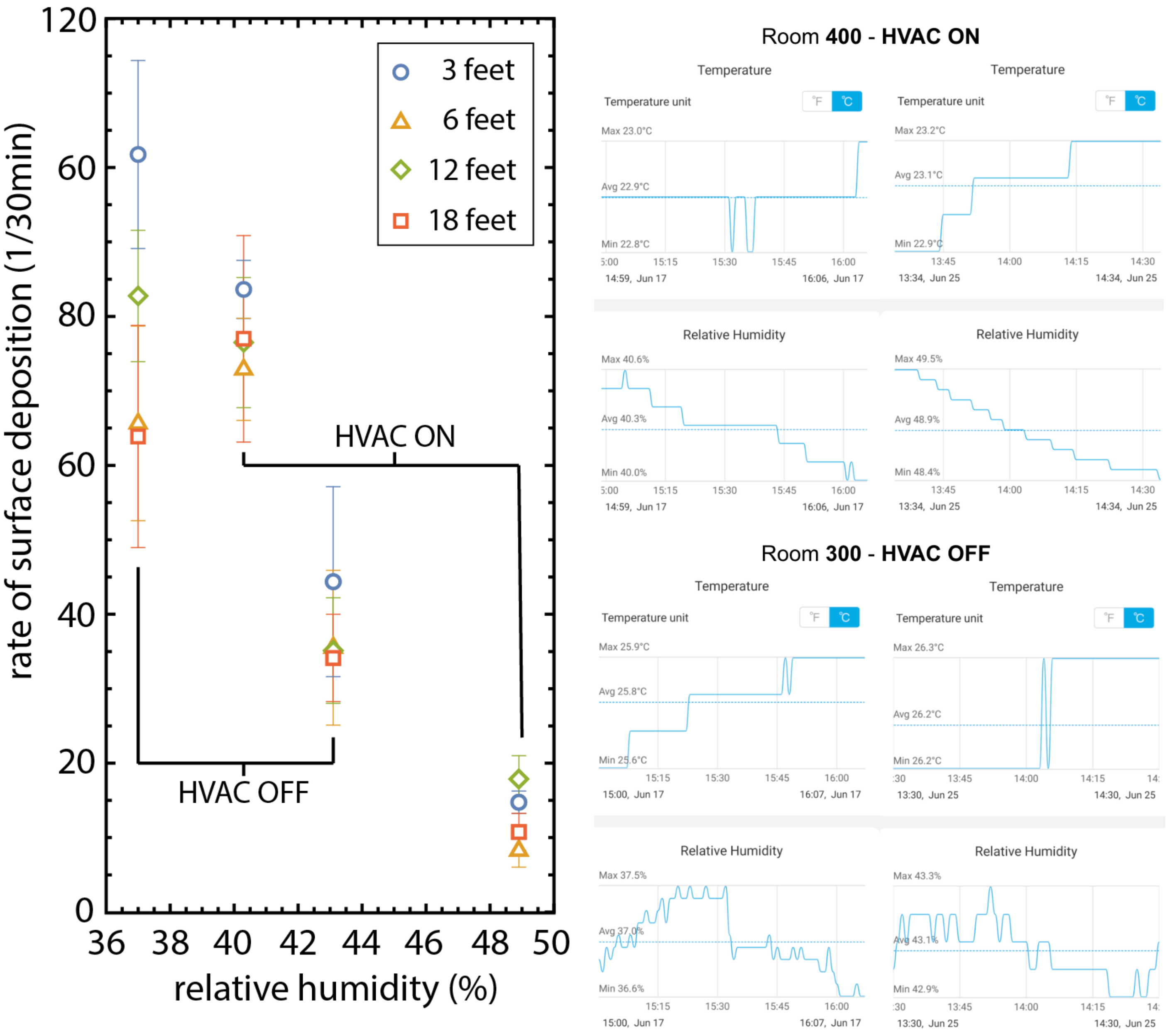

Figure S5: Horizontal surface deposition experiments with and without HVAC. We placed two sets of two plates horizontally on the bench surface, three feet apart, on the left and right side of the nebulizer, and a set of two vertical plates in the center. One plate in each set was exposed for 30 minutes, and the other for 60 minutes (See **Table S3**). In the room in which the HVAC was off we recorded temperatures of 25.8°C and 26.2°C, while in the room with HVAC on we recorded 22.9°C and 23.1°C. Relative humidity ranged between 36-50% (see detailed recordings in the right panel). Data points correspond to rates of PFUs in a 30-minute time window, averaged over the two replicates and two timepoints, for $3{\times}10^8$ total phage released. Error bars are S.E. We observe a decrease of PFUs with the increase in humidity, concurring with the results presented in the main text on vertical plates.

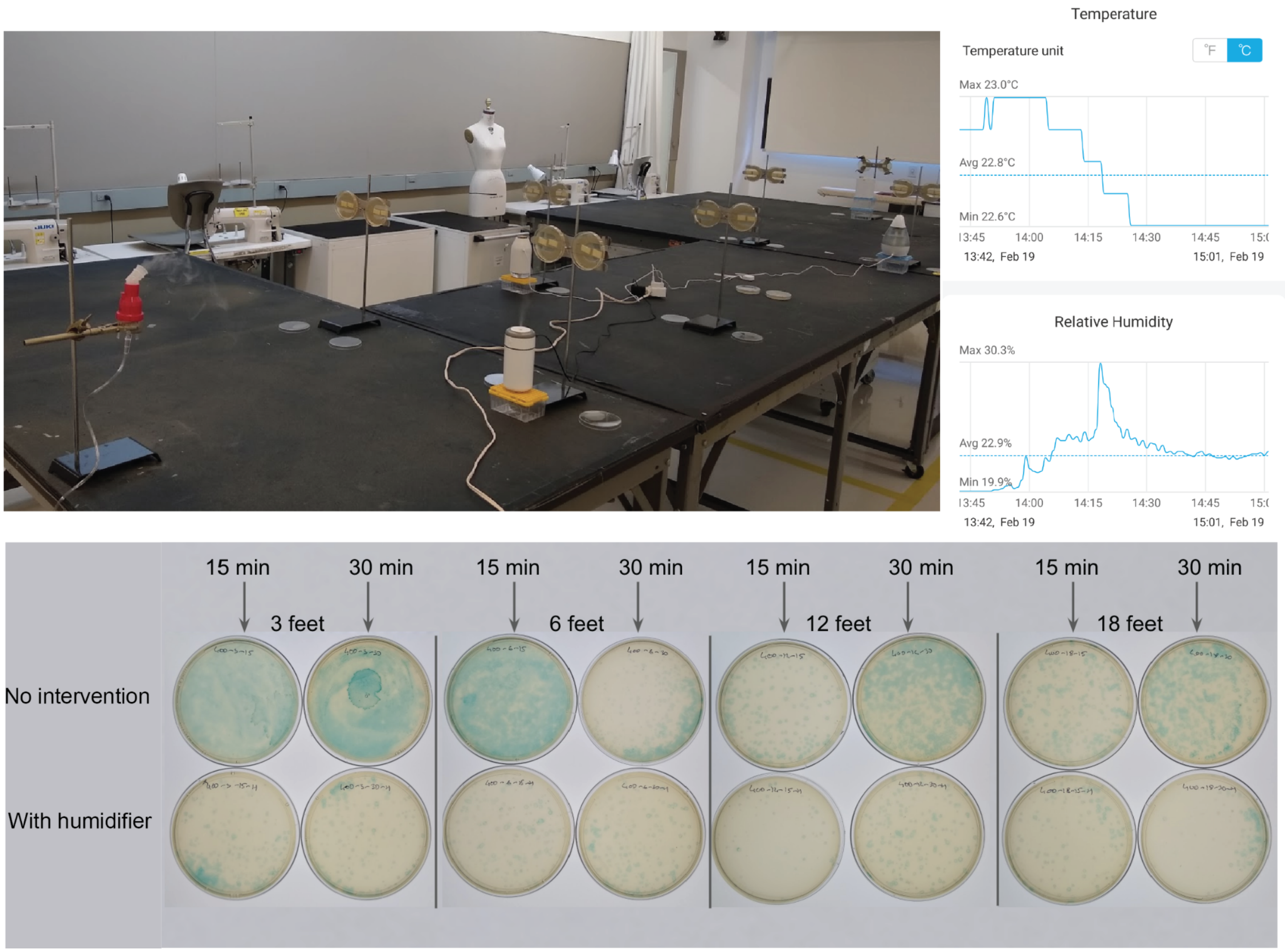

Figure S6: Experiments with personal humidifiers. We tested a number of personal humidifiers which we placed in front of one set of plates, while the other set at the same distance from the source had no intervention. We arranged the humidifiers in a zig-zag pattern to control for port-starboard preference. We started the humidifiers 15 minutes prior to starting phage aerosolization. We noticed a transient increase in relative humidity at a location of the measuring device, which was 9 feet away from the nebulizer (see temperature and humidity recordings in the top right panel). Plates with the humidifier show reduction of plaque counts when compared to the plates without intervention within the same experiment.

Table S1: PFU counts collected in **Room 300** (superscript C next to an entry means that plate had a contaminant, the number indicates how many contaminants were observed)

Date: April 21st, 2021		Titer $9x10^7$ PFU/mL		Volume nebulized: 5.6 mL	Relative Humidity: 37.2%	Average Temp.: 22.8°C	Total phage $5x10^8$	Controls: 0, 0
**Replicates**	**L**	**R**	**L**	**R**	**L**	**R**	**L**	**R**
**Distance/Time**	**3 feet**		**6 feet**		**12 feet**		**18 feet**	
**15 min**	218	367	16	68	14	11	22	1
**30 min**	37	140	18	84	1	1	10	4
**45 min**	51	136	166	63	26	42	3	13
**60 min**	73	128	107[C1]	319	80	122	99	1

Date: May 5th 3rd, 2021		Titer $9x10^7$ PFU/mL		Volume nebulized: 4.2 mL	Relative Humidity: 44.7%	Average Temp.: 22.5°C	Total phage $4x10^8$	Controls: 0, 0
**Replicates**	**L**	**R**	**L**	**R**	**L**	**R**	**L**	**R**
**Distance/Time**	**3 feet**		**6 feet**		**12 feet**		**18 feet**	
**15 min**	20	29	11	7	3	1	6	2
**30 min**	4	8	7	21	3	8	6	13
**45 min**	10	9	6	15	4	7	3	5
**60 min**	6	25	16	11	2	18	6	12

Date: June 3rd, 2021		Titer $8x10^6$ PFU/mL		Volume nebulized: 5.2 mL	Relative Humidity: 55.6%	Average Temp.: 23.1°C	Total phage $4x10^7$	Controls: 0, 0
**Replicates**	**L**	**R**	**L**	**R**	**L**	**R**	**L**	**R**
**Distance/Time**	**3 feet**		**6 feet**		**12 feet**		**18 feet**	
**15 min**	2	1	1	0	0	1	1	0
**30 min**	1	1	2	0	0	0	1	1
**45 min**	0	2	1	1	4	0	0	0
**60 min**	1	2	1	2	0	0	0	0

Table S2: PFU counts collected in **Room 400** (superscript ^{C} next to an entry means that plate had a contaminant; TNTC - too numerous to count -- in those cases we used 300 as a reference value)

Date: April 21st, 2021		Titer $9x10^7$ PFU/mL		Volume nebulized: 6.7 mL	Relative Humidity: 38.2%	Average Temp.: 22.7°C	Total phage $6x10^8$	Controls: 0, 0
**Replicates**	**L**	**R**	**L**	**R**	**L**	**R**	**L**	**R**
**Distance/Time**	**3 feet**		**6 feet**		**12 feet**		**18 feet**	
**15 min**	15	37	63	57	5	12	15	37
**30 min**	27	25	118	12	28	15	7	27
**45 min**	60	46	36	51	21	25	22	75
**60 min**	35	295	58	26	29	36	73	70

Date: April 22nd, 2021		Titer $7x10^7$ PFU/mL		Volume nebulized: 5.5 mL	Relative Humidity: 24.8%	Average Temp.: 22.8°C	Total phage $4x10^8$	Controls: 0, 0
**Replicates**	**L**	**R**	**L**	**R**	**L**	**R**	**L**	**R**
**Distance/Time**	**3 feet**		**6 feet**		**12 feet**		**18 feet**	
**15 min**	51	46	40	8	4	30	11	29
**30 min**	184	102	67	47	21	55	28	46
**45 min**	121	176	51	79	47	70	37	107
**60 min**	TNTC	TNTC	320	202	28	26	88	199

Date: May 5th, 2021		Titer $1.5x10^8$ PFU/mL		Volume nebulized: 6.4 mL	Relative Humidity: 43.7%	Average Temp.: 22.7°C	Total phage $10x10^8$	Controls: 0, 0
**Replicates**	**L**	**R**	**L**	**R**	**L**	**R**	**L**	**R**
**Distance/Time**	**3 feet**		**6 feet**		**12 feet**		**18 feet**	
**15 min**	9	9	4	3	2	0	1	0
**30 min**	4	9	5	10	8	5	9	1
**45 min**	20	17	38	16	5	5	22	3
**60 min**	92	11	19	18	15	15	7	4

Date: June 3rd, 2021		Titer $8x10^6$ PFU/mL		Volume nebulized: 5.8 mL	Relative Humidity: 52.7%	Average Temp.: 22.9°C	Total phage $5x10^7$	Controls: 0, 0
**Replicates**	**L**	**R**	**L**	**R**	**L**	**R**	**L**	**R**
**Distance/Time**	**3 feet**		**6 feet**		**12 feet**		**18 feet**	
**15 min**	0	0	0	0	0	0	0	0
**30 min**	0	0	0	0	0	0	0	0
**45 min**	0	0	0	0	0	0^{C1}	0	0
**60 min**	0^{C1}	0	0	0	0	0	0	0

Table S3: PFU counts collected in surface deposition experiments in room 300 while the HVAC was off and in room 400 while the HVAC was on. (L - horizontal plates on the port side, R - horizontal plates on the starboard side, V - vertical plates in the center)

**Room 400 HVAC ON**	Date: June 17th, 2021		Titer $5x10^7$ PFU/mL		Volume nebulized: 6mL		RH: 40.3%, T: 22.9°C			Total phage $3x10^8$		Controls: 0, 0
**Plate placement**	**L**	**V**	**R**	**L**	**V**	**R**	**L**	**V**	**R**	**L**	**V**	**R**
**Distance/Time**	**3**			**6**			**12**			**18**		
**30**	76	1	92	73	1	72	95	2	73	64	24	49
**60**	177[C1]	9	156	180	9	113	168	27	108	227	63	163

**Room 300 HVAC OFF**	Date: June 17th, 2021		Titer $5x10^7$ PFU/mL		Volume nebulized: 5.6mL		RH: 37%, T: 25.8°C			Total phage $3x10^8$		Controls: 0, 0
**Plate placement**	**L**	**V**	**R**	**L**	**V**	**R**	**L**	**V**	**R**	**L**	**V**	**R**
**Distance/Time**	**3**			**6**			**12**			**18**		
**30**	115	34	65[C1]	34	8	55	61	9	103[C1]	66	20	26
**60**	242	12	212[C1]	164	34	183	177	15	157	129[C1]	85	198[C1]

**Room 400 HVAC ON**	Date: June 25th, 2021		Titer $4x10^7$ PFU/mL		Volume nebulized: 7mL		RH: 48.9%, T: 23.1°C			Total phage $3x10^8$		Controls: 0, 0
**Plate placement**	**L**	**V**	**R**	**L**	**V**	**R**	**L**	**V**	**R**	**L**	**V**	**R**
**Distance/Time**	**3**			**6**			**12**			**18**		
**30**	16	7	11	13	1	4	16	1	13	17	1	6
**60**	36	3	28	10	2	22	31	0	54[C1]	15	5	25

**Room 300 HVAC OFF**	Date: June 25th, 2021		Titer $4x10^7$ PFU/mL		Volume nebulized: 6.8mL		RH: 43.1%, T: 26.2°C			Total phage $3x10^8$		Controls: 0, 0
**Plate placement**	**L**	**V**	**R**	**L**	**V**	**R**	**L**	**V**	**R**	**L**	**V**	**R**
**Distance/Time**	**3**			**6**			**12**			**18**		
**30**	14	30	36	28	1	11	18	3	33	43	4	20
**60**	148	20	107	87	3	119	74	63	105	58	38	89
